# 2385. Factors associated with delay of completion of a two-dose COVID-19 vaccine primary series among servicemembers in the Military Health System

**DOI:** 10.1093/ofid/ofad500.2005

**Published:** 2023-11-27

**Authors:** Erica Sercy, Laveta Stewart, Megan Clare Craig-Kuhn, Caryn Stern, Brock Graham, Amber Michel, Edward Parmelee, Stacy Shackelford, Simon Pollett, Timothy Burgess, David R Tribble, David R Tribble

**Affiliations:** Infectious Disease Clinical Research Program, Department of Preventive Medicine and Biostatistics, Uniformed Services University of the Health Sciences; Henry M. Jackson Foundation for the Advancement of Military Medicine, Inc., Washington, District of Columbia; Infectious Disease Clinical Research Program, Henry Jackson Foundation, Bethesda, Maryland; Infectious Disease Clinical Research Program, Department of Preventive Medicine and Biostatistics, Uniformed Services University of the Health Sciences; Henry M. Jackson Foundation for the Advancement of Military Medicine, Inc., Washington, District of Columbia; Joint Trauma System, JBSA Fort Sam Houston, Texas; Joint Trauma System, JBSA Fort Sam Houston, Texas; Infectious Disease Clinical Research Program, Department of Preventive Medicine and Biostatistics, Uniformed Services University of the Health Sciences; Henry M. Jackson Foundation for the Advancement of Military Medicine, Inc., Washington, District of Columbia; Infectious Disease Clinical Research Program, Department of Preventive Medicine and Biostatistics, Uniformed Services University of the Health Sciences, Bethesda, Maryland; Joint Trauma System, JBSA Fort Sam Houston, Texas; Infectious Disease Clinical Research Program, Department of Preventive Medicine and Biostatistics, Uniformed Services University of the Health Sciences, Bethesda, MD, USA, Bethesda, Maryland; Infectious Disease Clinical Research Program, Department of Preventive Medicine and Biostatistics, Uniformed Services University of the Health Sciences, Bethesda, MD, USA, Bethesda, Maryland; Uniformed Services University of the Health Sciences, Bethesda, Maryland; Uniformed Services University of the Health Sciences, Bethesda, Maryland

## Abstract

**Background:**

Efficacy of a two-dose COVID-19 vaccine series at preventing infection is ∼50-80% after the first dose and ∼95% following the second, if completed on the recommended schedule. Although >90% of active-duty servicemembers (SM) completed a primary series, understanding timing of doses as recommended among Military Health System (MHS) beneficiaries is vital to prevent COVID-19 outbreaks.

**Figure:**
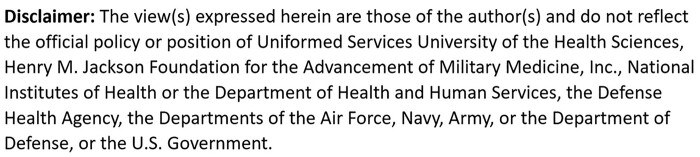

**Methods:**

Active-duty and National Guard/Reserve on active-duty SM aged ≥17 years who were beneficiaries of the MHS during 1/1/2020-6/30/2022 and completed a two-dose primary series were assessed for timing of the vaccines (stratified as early, grace period, recommended, and late) and associated demographic factors. SARS-CoV-2 infection (laboratory confirmed or ICD-10 diagnosis) prior to initiating the primary series, between the two doses, or after completion of the primary series was also assessed. Descriptive statistics (chi-square) were used to identify associations with timing of the second primary dose.

**Results:**

Among 1,426,436 SM, 97.9% were aged 17-49, 81.4% were male, 69.8% were white, and 35.6% served in the Army (Table 1). Overall, 92.7% received their second primary dose in the recommended or grace periods, and 7.3% were late. Timing of the first and second vaccine doses showed peaks in the months following the vaccine EUA, as well as after the DoD vaccine mandate. SM who had a COVID diagnosis between their two primary doses were more likely to receive a late second dose, although the diagnosis was likely not the cause for the late second dose, as were those who received their first dose during the pre-mandate period. SM receiving a late second dose were more likely to be younger, in the Air Force or Army, and reside in HHS regions 4, 5, 6, 7, and 8.

**Table 1.**
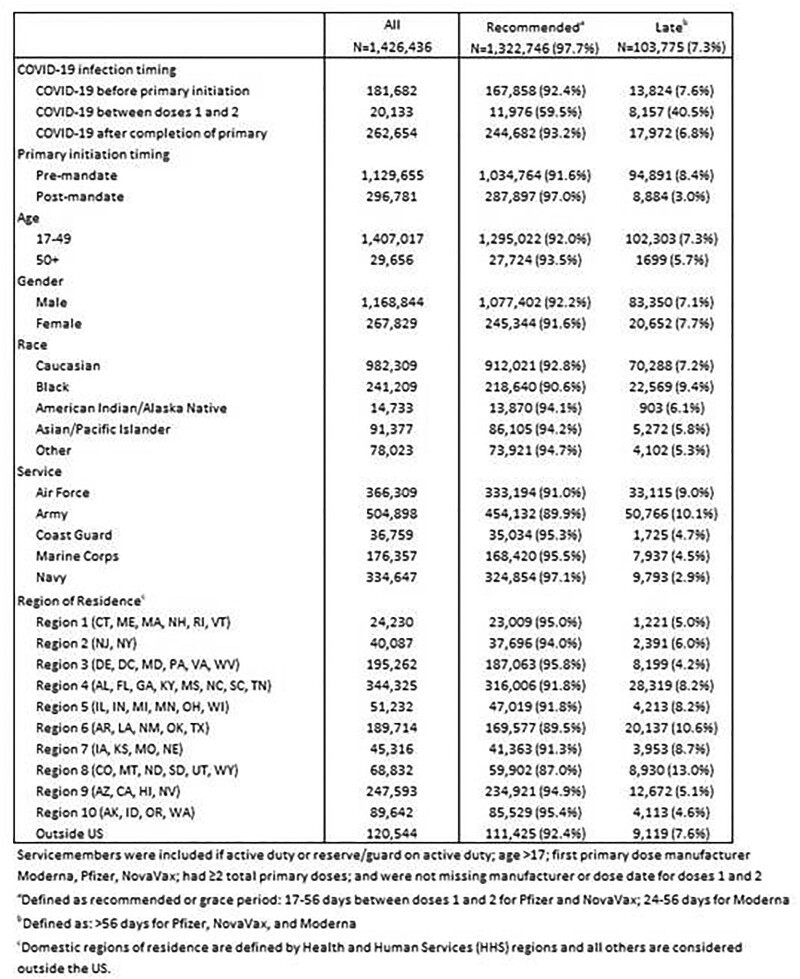
Demographics and COVID-19 status by timing of the second dose in a two-dose COVID-19 vaccine series.

**Conclusion:**

Most of the population received their second primary dose on the recommended schedule or earlier. However, SM in the Air Force and Army were more likely to receive their second dose later than recommended, as were those who lived in the South, West, and Midwest and those who had COVID-19 infection between their primary doses. Future research will investigate adverse disease outcomes associated with a delayed second dose in the primary series, as well as potential interactions between factors predicting delayed second dose uptake.

**Disclosures:**

**Simon Pollett, MBBS**, AstraZeneca: The IDCRP and the Henry M. Jackson Foundation (HJF) were funded to conduct an unrelated phase III COVID-19 monoclonal antibody immunoprophylaxis trial **Timothy Burgess, MD, MPH**, AstraZeneca: The IDCRP and the Henry M. Jackson Foundation (HJF) were funded to conduct an unrelated phase III COVID-19 monoclonal antibody immunoprophylaxis trial

